# SCM: A method to improve network service layout efficiency with network evolution

**DOI:** 10.1371/journal.pone.0189336

**Published:** 2017-12-21

**Authors:** Qi Zhao, Chuanhao Zhang, Zheng Zhao

**Affiliations:** 1 Computer Science and Technology College, Jilin University, Changchun, Jilin, China; 2 Department of Public Security Technology, Railway Police College, Zhengzhou, Henan, China; 3 National Digital Switching System Engineering & Technological R&D Center, Zhengzhou, Henan, China; 4 Department of Network Engineering, Zhengzhou Science and Technology Institute, Zhengzhou, Henan, China; Xiamen University, CHINA

## Abstract

Network services are an important component of the Internet, which are used to expand network functions for third-party developers. Network function virtualization (NFV) can improve the speed and flexibility of network service deployment. However, with the evolution of the network, network service layout may become inefficient. Regarding this problem, this paper proposes a service chain migration (SCM) method with the framework of “software defined network + network function virtualization” (SDN+NFV), which migrates service chains to adapt to network evolution and improves the efficiency of the network service layout. SCM is modeled as an integer linear programming problem and resolved via particle swarm optimization. An SCM prototype system is designed based on an SDN controller. Experiments demonstrate that SCM could reduce the network traffic cost and energy consumption efficiently.

## Introduction

Middleboxes [1], hardware-based network services, are widely deployed in the Internet and being recognized as important components of networks, such as their uses as firewalls, intrusion detection systems/intrusion prevention systems (IDS/IPS), load balancers, agencies, network address translators (NAT), and wide area network (WAN) optimizers etc. Service chains [2] are created by combining these service instances according to network policies and user requirements. Service chains satisfy various needs of users and provide value-added services to networks.

The flexibility and expansibility of the service deployment can be improved by network function virtualization (NFV) [3–5]. In NFV, special hardware middleboxes are replaced by virtual services, thus service chain customization and dynamical creation can be achieved. In addition, software defined networks (SDN) [6–8] are widely used to orchestrate network services according to policies and control traffic to pass special service chains [9–12]. The "SDN+NFV" network paradigm [13] provides a flexible, scalable, and adaptable architecture for deploying virtual services and creates new opportunities for service chain management.

Based on the "SDN+NFV" architecture, there has been extensive researches about service chain deployment [12, 14–17]. In these studies, service chains are deployed with consideration of the quality of service, resource allocation, and network security. However, these service chain placement approaches only consider the current network static status, and the changes of the network state are neglected. With network evolution, some old flows may disappear and new flows may arise. Therefore, the status of network changes and the service chain deployment may lose optimality with network evolution, resulting in a waste of network resources and energy. Moreover, the quality of services will decrease due to the degraded service chain layout.

This problem is difficult to resolve in service chain deployment due to the limited ability to anticipate the evolution of network states. However, if the deployment of service chains is dynamic throughout their life cycles, the network resource allocation can be adjusted dynamically to adapt to the changing network status. In this paper, a service chain migration (SCM) framework is proposed to address this problem, in which service chains are migrated dynamically to adapt to network evolution. SCM satisfies policy demands and improves the effectiveness of the network service instance layout. We model SCM as an integer linear programming (ILP) problem, and particle swarm optimization (PSO) [18] is used to solve the SCM problem.

The contributions of this work are as follows.

1. Two scenarios are shown to illustrate that the efficiency of the network resource layout decreases with network evolution and that service chain migration is utilized to improve the situation.
2. An SCM framework is proposed to optimize the network resource layout. We model the SCM as an ILP problem, and a modified PSO algorithm is implemented to provide the optimal solution under certain network resource constraints.
3. An SCM prototype system is designed based on an SDN controller [19, 20], and the efficiency of the SCM is corroborated by several numerical simulations.

## Related work

In traditional networks, services are realized by middleboxes, which is a major way for third-party developers to expand network functions. Extensive research has been conducted in middlebox management. StEERING [12], a flexible and extensible middlebox management framework, can efficiently control network traffic to pass middleboxes. SIMPLE [14], a service policy execution layer, can instantiate network service policies into service chains, where load balance and costs of network are considered in the service chain construction. Stratos [9], a network service orchestration framework, considers the changes of network load, and the service chains are constructed efficiently. The above three kinds of frameworks put effort into the service chains to optimize the trafic or load balance. However, the network evolution is not considered, which may limit the effectiveness of the network. Similar to the work above, SCM is also designed to manage network service, but the goal of SCM is to optimize service layouts for network evolution. SCM dynamically adjusts the deployed service chains, and the network resource allocation is optimized according to network state. In additoin, more benefits can be obtained in SCM, such as traffic optimization, energy saving and lower network cost.

Virtual machine migration has been proven to have the ability to increase the resource utilization rate and reduce energy consumption. A dynamic virtual machine migration method is proposed in [21], where migration traffic, available bandwidth, and balance of bandwidth capacity are considered during migration. In [22], virtual machine migration and network routing optimization are utilized to reduce the network energy consumption in the data center. Nidhi et al. [23] proposed an energy-aware virtual machine migration method in a cloud environment to improve the efficiency and decrease the energy consumption of the network. For energy saving, a novel placement selection policy of live virtual machine (VM) migration named PS-ES [24] is proposed. This method combines particle swarm optimization algorithm and simulated annealing to obtain the placement selection policy of the live VM migration. From the point of VM controller, Wen and et al. propose LS-STR [25], an adaptive controller for data center, based on the least square self-tuning regulator. The method can adjust VM resources dynamically and reduce the energy cost. Instead of migrating a single virtual machine or service, as described in the studies above, we migrate service chains to further optimize traffic cost and energy consumption. More specifically, SCM migrates multiple virtual machines periodically and the best migration strategy is determined by solving a ILP problem.

## Motivating scenarios

For convenience, the abbreviations used throughout this paper are listed in Table 1.

**Table 1:** Abbreviations.

Abbreviation	Full name
NFV	Network Function Virtualization
SDN	Software Defined Network
SCM	Service Chain Migration
VM	Virtual Machine
ILP	Integer Linear Programming
PSO	Particle Swarm Optimization
OF	OpenFlow
EA	Evolutionary Algorithm

Since the demands of users are dynamic, new policies are added in the network and outdated policies are deleted. With the network evolution, the fixed layout of service instances may lower the utilization efficiency of network resources. However, by migrating service chains, the efficiency of resource layout may be improved. In this paper, two scenarios are shown where service chain migration can reduce traffic cost and energy consumption, respectively.

**Fig 1:**
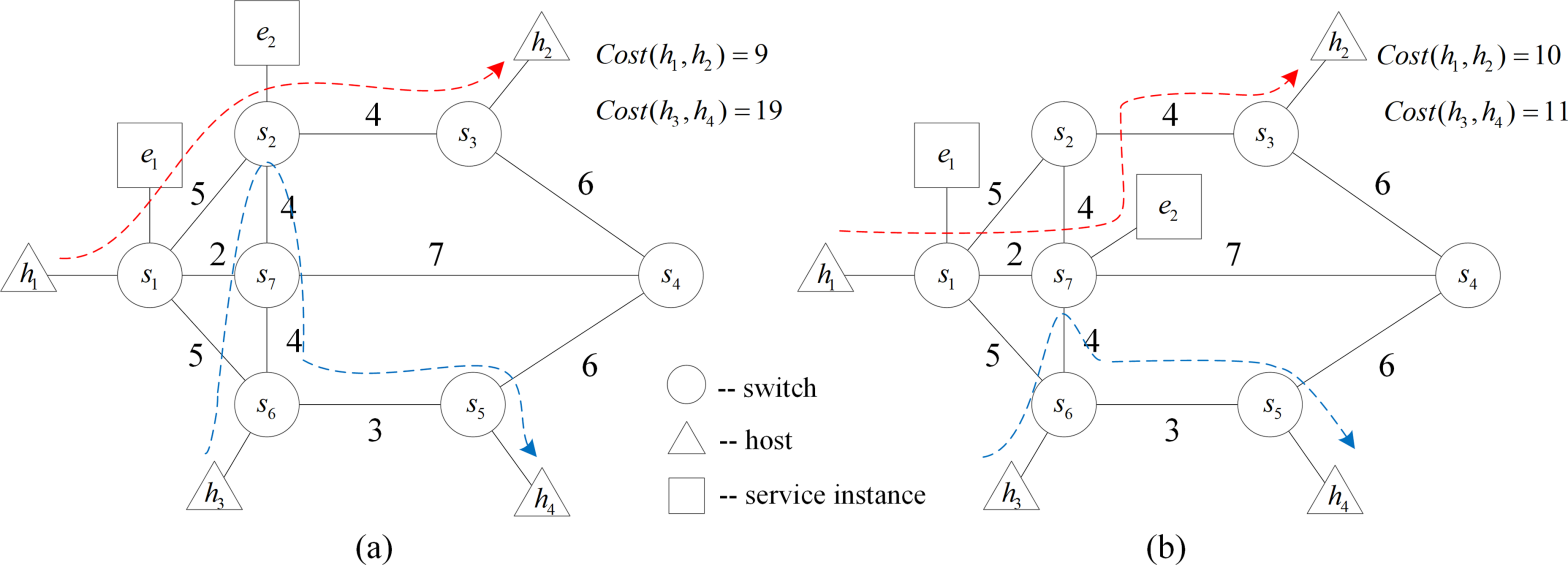
A scenario for reducing traffic cost.

**Fig 2:**
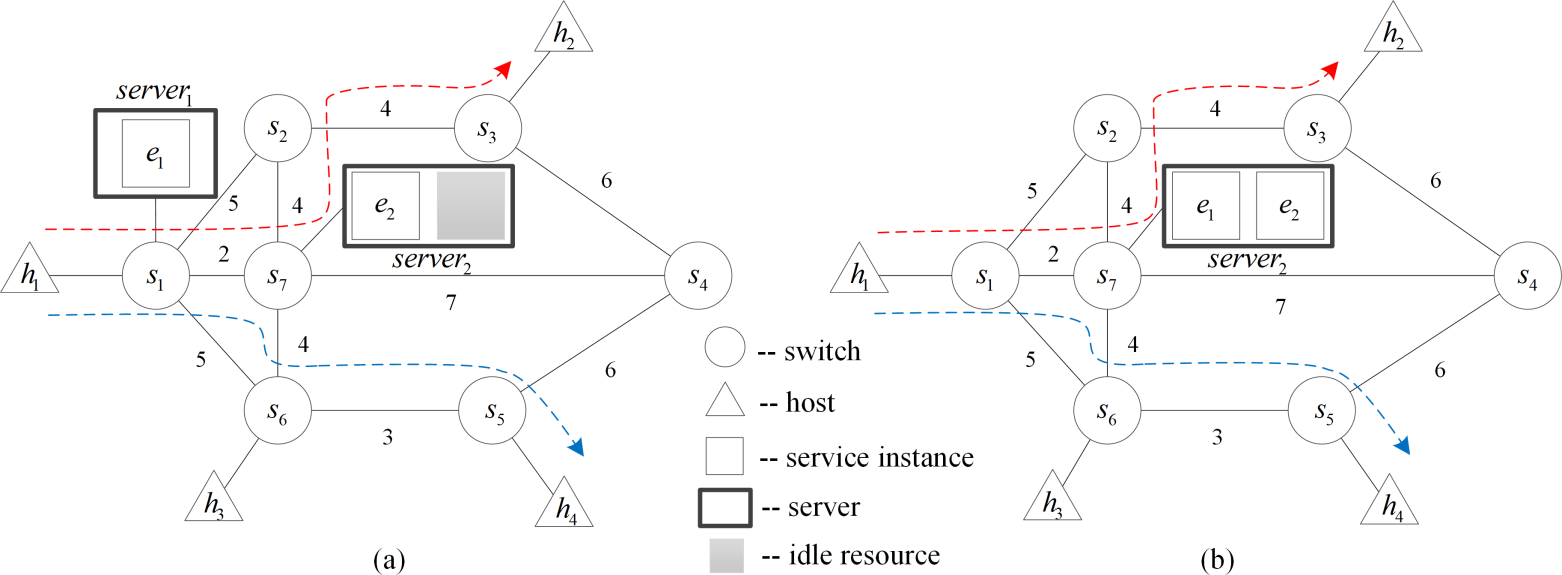
A scenario for reducing energy consumption.

1. **To optimize service layout and reduce traffic cost**: The network state evolution may lower the efficiency of the service layout. As shown in Fig 1(A), there are two service instances *e*_1_ and *e*_2_ in the network, and the traffic cost on each link is shown in the figure. Two policies are deployed successively in the network. Policy 1: The communication between hosts *h*_1_ and *h*_2_ needs two service instances *e*_1_ and *e*_2_. The traffic cost is given by *Cost*(*h*_1_,*h*_2_) = 9. Policy 2: The hosts *h*_3_ and *h*_4_ need service instance *e*_2_ during communication, and the traffic cost is *Cost*(*h*_3_,*h*_4_) = 19, which is larger. If the service instance *e*_2_ is migrated to the server connected with *s*_7_, the service chains will be migrated accordingly. The paths *h*_1_ → *h*_2_ and *h*_3_ → *h*_4_ will be adjusted as shown in Fig 1(B). In this circumstance, *Cost*(*h*_1_,*h*_2_) = 10 and *Cost*(*h*_3_,*h*_4_) = 11, and the total traffic cost will be decreased.
2. **To reduce the number of running servers and save energy:** As the network evolves over time, one server may carry only one or very few running service instances, which leads to a waste of energy. As shown in Fig 2(A), the current routing paths *h*_1_ → *h*_2_ and *h*_1_ → *h*_4_ are marked by dashed arrows, and both policies need the two service instances *e*_1_ and *e*_2_. The server *server*_1_ carries only one service *e*_1_, while *server*_2_ still has sufficient resource to hold one more service instance. Obviously, energy consumption will be reduced if we migrate service *e*_1_ to *server*_2_ (Fig 2(B)) and turn off *server*_1_.

## Modeling

In order to solve the problem of low efficiency of the resource layout caused by network evolution, service chains are considered to be migrated periodically under constraints. Service chain migration means migrating service instances in server chains from a server to another position without violating the policy demand. We first present relevant definitions, and then the service chain migration model is proposed. Finally, we present an intelligent optimization algorithm to solve the model.

### Basic definitions

**Service instance:** A service instance is an instantiated service equipment, which can be represented as a 3-unit set: *e* = <*ID*,*T*,*V*>, where *ID* is the unique identification of a service instance, *T* is the type of service, and *V* is the amount of data to be transferred for migrating this service instance. Let *E* = {*e*_1_,*e*_2_,⋯,*e*_*h*_} denote the set of all service instances deployed in the network.

**Service chain:** A service chain is a group of sequential service instances, which can satisfy user’s specific demands for communication. Let $E_{p}=\{e_{1},\cdots ,e_{j^{\prime }},\cdots ,e_{j^{\prime \prime }}\}$ denote a service chain.

**Policy:** A policy is defined as $p≜(O_{p},E_{p},D_{p})$, where *O*_*p*_ denotes the policy target, i.e., the specific traffic processed by poli*cy p*, $E_{p}$ denotes the service chain that processes traffic *O*_*p*_. The number of service instances in $E_{p}$ is denoted by |*p*|. And $D_{p}≜(d_{p}^{b},d_{p}^{c})$ is the demand set of policy *p*, where $d_{p}^{b}$ denotes the bandwidth demand, and $d_{p}^{c}$ denotes the path cost demand.

**Server:** A server is a running platform for virtual service instances, which can provide storage and bandwidth resources for multiple service instances. Let *Z* = {*z*_1_,*z*_2_,⋯,*z*_*k*_} be the set of all servers in the network. For *z*_*s*_ ∈ *Z*, *w*_*s*_ denotes the maximum number of service instances that *z*_*s*_ can carry and *t*_*s*_ denotes the maximum bandwidth that *z*_*s*_ can process.

### SCM model

Compared with traditional virtual machine migration, the SCM model is more complex for two reasons. First, policy demands, such as the bandwidth and path cost, are taken into account in SCM. Second, the migration of one service instance in a service chain may affect the migration of other service instances in this service chain because of the correlations between them. We intent to get the best migration stragety under multiple factors to minimize the network cost. We define a utility value as the measurement of network cost of a migration and intent to get the minimum utility value of a migration under certain constrains, therefore, it can be regarded as a minimum value problem. As described below, the objective function and constraints are considered to be linear in our study. In addition, the solutions of SCM belongs to the integer domain. Therefore, we modeled SCM as a problem of ILP.

For a migration *M*, the optimization goal of SCM is shown in Eq (1):

$$\mathrm{SCM}(M)\;\min\;Utility=\alpha_{1}\beta_{1}U+\alpha_{2}\beta_{2}R+\alpha_{3}\beta_{3}Q$$ (1)

$$\alpha_{1}+\alpha_{2}+\alpha_{3}=1$$ (2)

where *Utility* is the utility value of migration *M*; *U* is the traffic cost of the whole network policies; *R* is the number of running servers; *Q* is the migration cost of the service instance; *α*_1_,*α*_2_,*α*_3_ are the weights of *U*, *R*, and *Q*, respectively, whose total sum is 1; and *β*_1_,*β*_2_,*β*_3_ are the parameters that unify the dimensions of *U*, *R* and *Q*.

$$U=\sum_{p\in P}^{}\sum_{\begin{array}{c} j\in [0,\left|p\right|]\\ e_{i^{\prime }}=E_{p}[j];e_{i}=E_{p}[j+1] \end{array}}^{}\sum_{z_{s^{\prime }}\in Z}\sum_{z_{s}\in Z}y_{i^{\prime }}^{s^{\prime }}y_{i}^{s}Cost\left(s^{\prime },s\right)f\left(p,i^{\prime },i\right)$$ (3)

The whole network traffic cost is calculated by Eq (3). The boolean variable $y_{i}^{s}$ indicates whether the service instance *e*_*i*_ is on server *z*_*s*_ after migration. *Cost*(*s*′,*s*) is the minimum path cost between server $z_{s^{\prime }}$ and *z*_*s*_. *f*(*p*,*i*′,*i*) is the traffic injecting into the subsequent service *e*_*i*_ from service $e_{i^{\prime }}$. $E_{p}[0]$ denotes the traffic source of policy *p*. The traffic from the source to the first service is the bandwidth demand $d_{p}^{b}$.

$$Q=\sum_{e_{i}\in E}^{}\sum_{z_{s}\in Z}^{}\sum_{z_{s^{\prime }}\in Z-\{z_{s}\}}^{}w_{i}l_{s^{\prime }s}x_{i}^{s^{\prime }}m_{i}^{s}$$ (4)

The migration of a service instance consumes network bandwidth resources. Eq (4) shows the bandwidth consumption caused by a service instance migration. The boolean variable $x_{i}^{s}$ indicates whether the service instance *e*_*i*_ is on server *z*_*s*_ in the original network. The boolean variable $m_{i}^{s}$ indicates whether *e*_*i*_ is migrating to *z*_*s*_. *w*_*i*_ is the volume of *e*_*i*_, and $l_{ss^{\prime }}$ is the shortest distance between server *z*_*s*_ and $z_{s^{\prime }}$.

A server with no service running on it will be turned off to reduce energy consumption. Eq (5) gives the number of running servers after migration, where $y_{i}^{s}$ indicates whether *e*_*i*_ is on *z*_*s*_ after migration.

$$R=\sum_{z_{s}\in Z}^{}\left(1-\prod_{e_{i}\in E}^{}\left(1-y_{i}^{s}\right)\right)$$ (5)

Based on the flow conservation principle, Eq (6) declares that the outflow of one service is equal to the inflow of the subsequent service. Network services may affect traffic (for example, a firewall may drop a part of traffic in a flow). In SCM, traffic influence factors are introduced to model the traffic influence caused by services. Let $\gamma_{p}^{j}$ be the traffic influence on the *j*-th service of policy *p*, which is calculated by $\gamma_{p}^{j}=\mathrm{outflow/inflow}$. For policy *p*, the traffic from the source to the first service is the bandwidth demand $d_{p}^{b}$, as shown in Eq (7).

$$\mathrm{For}\;\forall p\in P,\forall j\in [2,\left|p\right|],\;e_{i^{\prime }}=E_{p}[j-1],e_{i}=E_{p}[j],e_{i^{\prime \prime }}=E_{p}[j+1],$$

$$f\left(p,i,i^{\prime \prime }\right)=f\left(p,i^{\prime },i\right)\gamma_{p}^{j}$$ (6)

$$j=1,\;f(p,E_{p}[j-1],E_{p}[j])=d_{p}^{b}$$ (7)

Eq (8) shows the relationship between $x_{i}^{s}$, $y_{i}^{s}$, $m_{i}^{s}$, where $x_{i}^{s}\left(1-\sum_{z_{s^{\prime }}\in Z-\{z_{s}\}}^{}m_{i}^{s^{\prime }}\right)=1$ indicates the service instance *e*_*i*_ is running on the server *z*_*s*_ and has not been migrated; $\left(1-x_{i}^{s}\right)m_{i}^{s}=1$ indicates the service instance *e*_*i*_ is not running on *z*_*s*_ originally, but has been migrated to *z*_*s*_. $x_{i}^{s}$ and $m_{i}^{s}$ cannot be 1 at the same time. Eq (9) adds the constraint that the service instance *e*_*i*_ cannot be migrated to server *z*_*s*_ from *z*_*s*_ itself.

$$\forall e_{i}\in E,z_{s}\in Z,\;y_{i}^{s}=x_{i}^{s}\left(1-\sum_{z_{s^{\prime }}\in Z-\{z_{s}\}}^{}m_{i}^{s^{\prime }}\right)+\left(1-x_{i}^{s}\right)m_{i}^{s}$$ (8)

$$\forall e_{i}\in E,z_{s}\in Z,\;x_{i}^{s}m_{i}^{s}=0$$ (9)

Policy *p* demands that the cost from the source to the destination should not exceed $d_{p}^{c}$. Eq (10) indicates that the service chain should satisfy the cost demand after migration.

$$\forall p\in P,\;\sum_{\begin{array}{c} \;j\in [1,\left|p\right|]\\ e_{i}=E_{p}[j];e_{i^{\prime }}=E_{p}[j+1] \end{array}}^{}\sum_{z_{s}\in Z}\sum_{z_{s^{\prime }}\in Z}y_{i}^{s}y_{i^{\prime }}^{s^{\prime }}Cost\left(s,s^{\prime }\right)\leq d_{p}^{c}$$ (10)

For ∀*z*_*s*_ ∈ *Z*, its bandwidth is finite. Eq (11) indicates that the traffic injected from the network to the server *z*_*s*_ should not exceed its maximum bandwidth *t*_*s*_.

$$\forall z_{s}\in Z,\;\sum_{p\in P}^{}\sum_{\begin{array}{c} j\in [0,\left|p\right|]\\ e_{i}=E_{p}[j];e_{i^{\prime }}=E_{p}[j-1] \end{array}}^{}y_{i}^{s}f\left(p,i^{\prime },i\right)\leq t_{s}$$ (11)

For ∀*z*_*s*_ ∈ *Z*, the number of service instances running on the server *z*_*s*_ is restrained by the capacity of *z*_*s*_, as shown in Eq (12).

$$\forall z_{s}\in Z,\;\sum_{e_{i}\in E}^{}y_{i}^{s}\leq w_{s}$$ (12)

For one migration, one service instance can be migrated to no more than one server, which is indicated by Eq (13). Eq (14) shows the range of the variable $m_{i}^{s}$.

$$\forall e_{i}\in E,\;\sum_{z_{s}\in Z}^{}m_{i}^{s}\leq 1$$ (13)

$$\forall e_{i}\in E,z_{s}\in Z,\;m_{i}^{s}\in \{0,1\}$$ (14)

### Resolving algorithm

SCM(*M*) is a typical ILP problem, which is called the SCM problem in this paper. An exhaustive algorithm can be used to solve the SCM problem by traversing the solution space. However, the time consumption will be huge due to the large size of this problem. An alternative would be the heuristic search approach, such as the evolutionary algorithm (EA). EA has been studies extensively [26–28] and has shown to be suitable for solving real-world optimization problems with large solution space, containing multiple fields [29–32].We use PSO algorithm, a particularly interesting algorithm of EA family, to solve the SCM problem due to its advantage in convergence rate. Here, we adopt the binary PSO to solve the SCM problem as the solution space of the SCM problem is binary. The position and velocity parameters of the binary PSO are defined as follows.

**Position (*M*):** The boolean variable $m_{i}^{s}$ denotes whether service instance *e*_*i*_ is migrating to server *z*_*s*_ or not; the vector $\left(m_{i}^{1},m_{i}^{2},\cdots ,m_{i}^{\left|Z\right|}\right)$ indicates the migration of *e*_*i*_ (*e*_*i*_ ∈ *E*). *M*_*g*_ is position vector of the *g*-th particle in a particle swarm, which is defined as one migration of all service instances as shown in Eq (15), where |*Z*| is the number of servers and |*E*| is the number of service instances. Let *m*_*gd*_ be the *d*-th component of *M*_*g*_.

$$M_{g}=(m_{1}^{1},m_{1}^{2},\cdots ,m_{1}^{\left|Z\right|},\cdots ,m_{i}^{1},m_{i}^{2},\cdots ,m_{i}^{\left|Z\right|},\cdots ,m_{\left|E\right|}^{1},m_{\left|E\right|}^{2},\cdots ,m_{\left|E\right|}^{\left|Z\right|})$$ (15)

**Velocity (*V*):** The velocity vector $\left(v_{i}^{1},v_{i}^{2},\cdots v_{i}^{\left|Z\right|}\right)$ is used to migrate a service instance *e*_*i*_ into a better solution. In Eq (16), the vector *V*_*g*_ is the velocity of the *g*-th particle in the particle swarm. Based on the component $v_{i}^{s}$ in *V*_*g*_, the probability of component $m_{i}^{s}$ in *M*_*g*_ being 1 can be determined. Let *v*_*gd*_ be the *d*-th component of the velocity vector *V*_*g*_.

$$V_{g}=(v_{1}^{1},v_{1}^{2},\cdots ,v_{1}^{\left|Z\right|},\cdots ,v_{i}^{1},v_{i}^{2},\cdots ,v_{i}^{\left|Z\right|},\cdots ,v_{\left|E\right|}^{1},v_{\left|E\right|}^{2},\cdots ,v_{\left|E\right|}^{\left|Z\right|})$$ (16)

In the traditional binary PSO, the particle velocity and position updating equations are shown in Eqs (17)–(20). In Eq (17), *c*_1_ and *c*_2_ are constants, *ξ*,*η* are two independent random variables with uniform distribution on the interval of [0,1], *pBest*_*gd*_ is the *d*-th component in the historical optimal solution *pBest*_*g*_ of particle *g* (i.e. the local optimal solution), and *gBest*_*d*_ is the *d*-th component of the optimal solution *gBest* among all particles (i.e. the global optimum solution). *ω*(*t*) is the inertia weight in PSO. *m*_*gd*_ is the *d*-th component of *M*_*g*_ and *v*_*gd*_ is the *d*-th component of *V*_*g*_. In Eq (18), the random variable *random* has uniform distribution on the interval of [0,1]. In Eq (20), *ω*_max_ and *ω*_min_ are the maximum and minimum values of *ω*(*t*), respectively, *t* is the current number of iterations, and *MI* is the maximum number of iterations.

$$v_{gd}=\omega (t)v_{gd}+c_{1}\xi \left(pBest_{gd}-m_{gd}\right)+c_{2}\eta \left(gBest_{d}-m_{gd}\right)$$ (17)

$$m_{gd}=\left\{ \begin{array}{l} 1,\;if\;random<Sig(v_{gd})\\ 0,\;else \end{array}\right.$$ (18)

$$Sig(v_{gd})=\frac{1}{1+\exp(-v_{gd})}$$ (19)

$$\omega \left(t\right)=\omega_{\max}-\frac{\omega_{\max}-\omega_{\min}}{MI}\times t$$ (20)

From Eq (18), it can be known that the value of boolean variable *m*_*gd*_ have randomness. Therefore, the position updating of one particle may cause one service instance to be migrated to multiple servers, which violates the constraint in Eq (13). Thus, it is difficult to find feasible solutions. To solve this problem, the position updating equation Eq (18) is modified with consideration to the constraint in Eq (13).

In the velocity vector *V*_*g*_, the |*Z*| components $\left(v_{i}^{1},v_{i}^{2},\cdots ,v_{i}^{\left|Z\right|}\right)$ represent the migration of the service instance *e*_*i*_, and $Sig(v_{i}^{s})$ represents the probability that $m_{i}^{s}$ is 1 among the |*Z*| components $\left(m_{i}^{1},m_{i}^{2},\cdots ,m_{i}^{\left|Z\right|}\right)$ in the position vector *M*_*g*_. Let:

$$\lambda_{i}^{s}=Sig(v_{i}^{s})$$ (21)

$$\lambda_{i}=(\lambda_{i}^{1},\lambda_{i}^{2},\cdots ,\lambda_{i}^{\left|Z\right|})$$ (22)

Eq (22) represents the probabilities that a service instance *e*_*i*_ is migrated to servers in *Z*. Since a service instance *e*_*i*_ can be migrated to only one server, there is at most one component $m_{i}^{s}$ in $\left(m_{i}^{1},m_{i}^{2},\cdots ,m_{i}^{\left|Z\right|}\right)$ with a value of 1 (1 ≤ *s* ≤ |*Z*|). If one component $m_{i}^{s}$ is 1, its probability is shown Eq (23).

$$\begin{array}{l} \lambda_{i}^{only(s)}=\left(1-\lambda_{i}^{1}\right)\times \left(1-\lambda_{i}^{2}\right)\times \cdots \times \left(1-\lambda_{i}^{s-1}\right)\times \lambda_{i}^{s}\times \left(1-\lambda_{i}^{s+1}\right)\times \left(1-\lambda_{i}^{\left|Z\right|}\right)\\ =\frac{\lambda_{i}^{s}}{1-\lambda_{i}^{s}}\prod_{k\in [1,\left|Z\right|]}\left(1-\lambda_{i}^{k}\right) \end{array}$$ (23)

If no component in $\left(m_{i}^{1},m_{i}^{2},\cdots ,m_{i}^{\left|Z\right|}\right)$ is equal to 1, i.e., all the components are 0, then the probability is marked as $\lambda_{i}^{only(0)}$ and calculated by Eq (24).

$$\begin{array}{l} \lambda_{i}^{only(0)}=\left(1-\lambda_{i}^{1}\right)\times \left(1-\lambda_{i}^{2}\right)\times \cdots \times \left(1-\lambda_{i}^{s-1}\right)\times \left(1-\lambda_{i}^{s}\right)\times \left(1-\lambda_{i}^{s+1}\right)\times \left(1-\lambda_{i}^{\left|Z\right|}\right)\\ =\prod_{k\in [1,\left|Z\right|]}\left(1-\lambda_{i}^{k}\right) \end{array}$$ (24)

There are |*Z*| + 1 cases for updating the position vector $\left(m_{i}^{1},m_{i}^{2},\cdots ,m_{i}^{\left|Z\right|}\right)$ under the constraint in Eq (13): one component is 1 while the other components are zero (|*Z*| cases) and all the components are 0 (1 case). The probability of each case can be calculated by Eq (25). Obviously, $\sum_{k\in [0,\left|S\right|]}\theta_{i}^{k}=1$.

$$\forall k\in [0,\left|Z\right|],\;\theta_{i}^{k}=\lambda_{i}^{only(k)}{\left(\sum_{s\in [0,\left|Z\right|]}\lambda_{i}^{only(s)}\right)}^{-1}$$ (25)

A probabilistic search procedure [33] is used to select $\theta_{i}^{k}$. If *k* = 0, then all components in $\left(m_{i}^{1},m_{i}^{2},\cdots ,m_{i}^{\left|Z\right|}\right)$ are 0. If 1 ≤ *k* ≤ |*Z*|, then $m_{i}^{k}=1$, while the other |*Z*|−1 components in $\left(m_{i}^{1},m_{i}^{2},\cdots ,m_{i}^{\left|Z\right|}\right)$ are all 0.

$$\mathrm{For}\;i\in [1,\left|E\right|],\;m_{i}^{k}=\left\{ \begin{array}{l} 1,\;if\;\sum_{s\in [0,k-1]}\theta_{i}^{s}\leq rd<\sum_{s\in [0,k]}\theta_{i}^{s}\\ 0,\;\mathrm{otherwise} \end{array}\right.,\;1\leq k\leq \left|Z\right|$$ (26)

The position updating of particle *g* is conducted in groups. First, the components of the position vector *M*_*g*_ are divided into |*E*| groups. Each group $\left(m_{i}^{1},m_{i}^{2},\cdots ,m_{i}^{\left|Z\right|}\right)$ corresponds to the migration of one service instance *e*_*i*_. $\left(m_{i}^{1},m_{i}^{2},\cdots ,m_{i}^{\left|Z\right|}\right)$ is updated based on Eq (26) (*rd* has uniform distribution on the interval of [0,1)). With this method, Eq (13) can be solved after the particle position vector is updated and the optimizing speed is improved by reducing the number of infeasible solutions.

To reduce the number of running servers, the service instances in small-load servers should be migrated with a high probability. Thus, Eq (21) is modified into Eq (27) by adding an encouragement factor, where *Threshold* is the server load threshold, *Enc*(*t*) is an encouragement function, as showed in Eq (28).

$$\lambda_{i}^{s}=\left\{ \begin{array}{ll} Sig(v_{i}^{s})+Enc(t)(1-Sig(v_{i}^{s})), & \mathrm{when}\;\mathrm{load}\;\mathrm{of}\;\mathrm{the}\;\mathrm{server}\;\mathrm{which}\;e_{i}\\ & \mathrm{is}\;\mathrm{located}\;\mathrm{is}\;\mathrm{lower}\;\mathrm{than}\;Threshold,\\ & \mathrm{and}\;\mathrm{load}\;\mathrm{of}\;z_{s}\;\mathrm{is}\;\mathrm{more}\;\mathrm{than}\;Threshold\\ Sig(v_{i}^{s}), & \mathrm{otherwise} \end{array}\right.$$ (27)

$$Enc(t)=\frac{1}{\theta e-1}\left({\left(\theta e\right)}^{\frac{t}{MI}}-1\right),t\in [1,MI]$$ (28)

**Fig 3:**
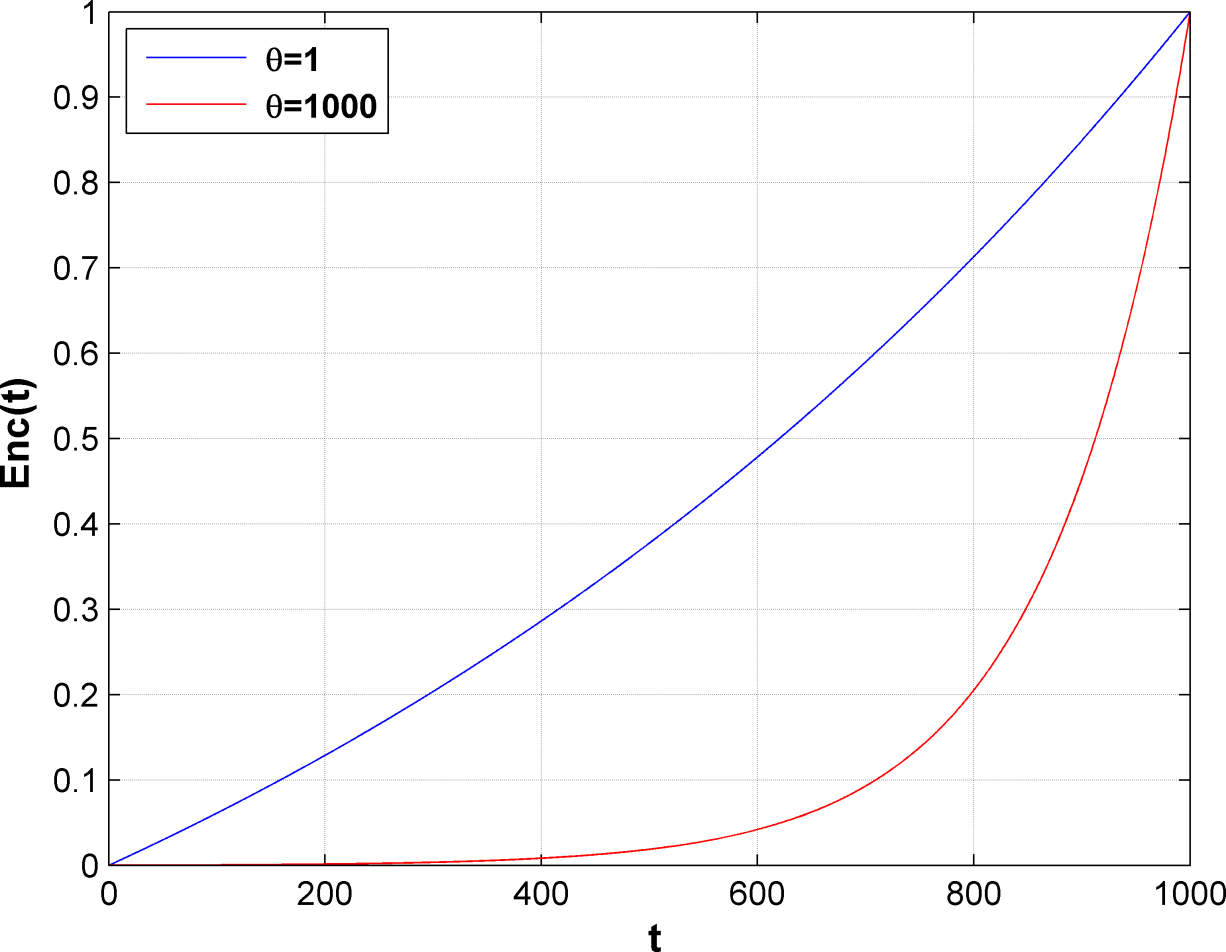
Encouragement function curves.

In Eq (28), *Enc*(*t*) changes with the number of iterations *t* and the parameter *θ*, as shown in Fig 3. We set *θ* = 1000. As shown in the figure, when the number of iterations is small, the instance migration is not encouraged, which enables PSO to escape from local optima and converge to the global optimum. When the number of iterations is large, it intends to migrate the instances in small-load servers to reduce the number of running servers.

To solve the SCM problem, Eq (1) is used as the fitness function in the PSO, marked as *fit*(*M*), where *M* indicates a possible migration. The constraints will be checked before computing the fitness function. If a migration *M* satisfies the constraints, *fit*(*M*) is computed; otherwise, *fit*(*M*) = +∞.

## Design of SCM Framework and Experiments

In this section, a design of the SCM framework is elaborated. Then, three metrics are proposed to evaluate the performance and cost of SCM. Finally, the performance and cost of SCM are compared and analyzed under different parameters.

### SCM framework

**Fig 4:**
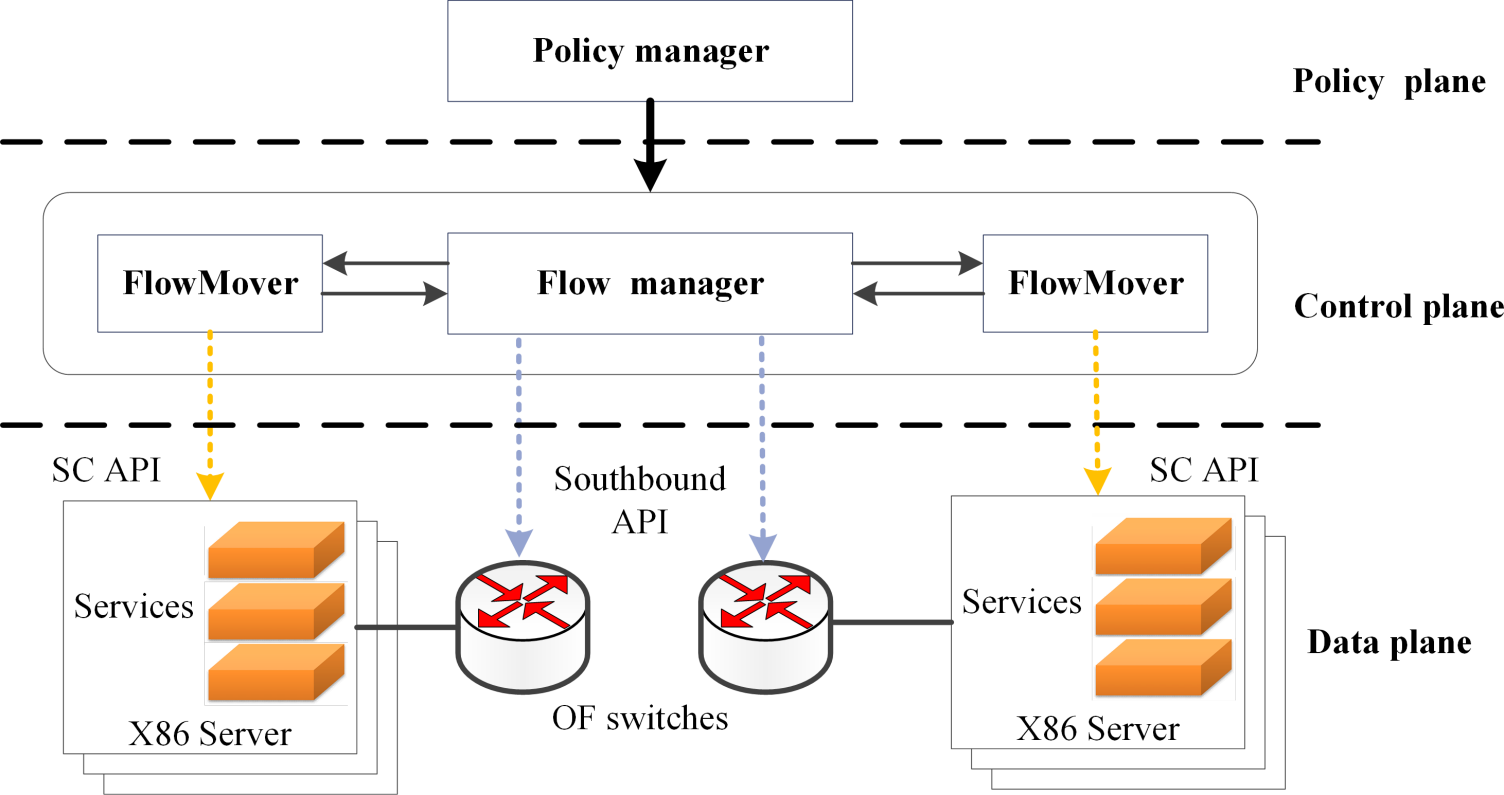
FlowMover framework.

We designed an SCM prototype system FlowMover based on the SDN controller. As shown in Fig 4, FlowMover consists of three planes: a policy plane, a control plane, and a data plane. The policy plane and the control plane are constructed based on the SDN controller. The Policy manager of the policy plane sends policies to the network. The control plane deploys the service instances according to the service chain policies and network state, and installs flows through the Fow manager (via the southbound API). The FlowMover in the control plane migrates the service chains periodically through service chain interface API (SC API).

In FlowMover, the traffic tag method proposed in [14] is applied. Specific traffic is tagged to pass a certain service chain based on the policy. There are many methods available for online virtual machine migration without interrupting services [34–36] and the virtual machine migration method proposed in [34] is adopted in SCM.

### Evaluation metrics

To evaluate the effectiveness and cost of SCM, we define three evaluation metrics: optimized rate of traffic cost *OE*_*U*_, optimized rate of number of running servers *OE*_*R*_, and migration rate of service instance *OE*_*Q*_, which can be calculated according to Eqs (29), (30) and (31), respectively. *U* and *U*′ denote the whole network traffic cost before and after service chain migration, *R* and *R*′ denote the numbers of running servers in the network before and after service chain migration, and *Q* and *Q*′ denote the numbers of all and migrated service instances in one service chain migration.

$$OE_{U}=\frac{U^{\prime }}{U}$$ (29)

$$OE_{R}=\frac{R^{\prime }}{R}$$ (30)

$$OE_{Q}=\frac{Q^{\prime }}{Q}$$ (31)

### Performance and cost assessment

Simulations of network evolution and service chain migration are implemented in MATLAB. BRITE [37], a network topology generator, is used in the experiments to generate a random topology with 100 nodes of Waxman model (*α* = 0.2, *β* = 0.15). In the experiment topology, the delay of each link in is considered as the cost of the link. Each network node is connected with a server, and all servers have the same capacity *w*_*s*_ and bandwidth resource *t*_*s*_. *t*_*s*_ is set to 50 unit traffic per unit time, and the threshold *Threshold* is set to ⌈*w*_*s*_/3⌉. The length of the service chain of policies has uniform distribution on the interval of [1,15], while the bandwidth demand of the policies has uniform distribution on the interval of [1,5]. The policy request follows a Poisson distribution with an average of 4 requests in 100 time units. The policy life cycle follows an exponential distribution with an expectation of 1000 time units. In the optimization goal of Eq (1), *β*_1_ = 1/U^*^, *β*_2_ = 1/R^*^, *β*_3_ = 1/Q^*^, where U^*^ and R^*^ denote the traffic cost and the number of running servers before service chain migration. Q^*^ represents the summation of service instance volumes multiplied by the average link length. For the service *p*[*j*] in the policy *p*, we set $\gamma_{p}^{j}=1$.

To simulate the evolution of the network state, the service chain deployment algorithm proposed in [38] is used in the experiments, and the service chains are deleted from the network when policies are outdated. The minimum cost between service instances is calculated by Dijkstra algorithm. The parameters setted in PSO are listed in Table 2. The simulation program runs on a computing platform with Intel Core i7 @3.10 GHz and 8G RAM.

**Table 2:** PSO parameter setting in the simulation.

Parameter	Value
Number of particles *N*_*P*_	30
Cognitive scaling parameter *c*_1_	2
Social scaling parameter *c*_2_	2
*ξ*,*η*	U(0,1)
Maximum inertia weight *ω*_max_	0.9
Minimum inertia weight *ω*_min_	0.4
Maximum number of iterations *MI*	1000

#### 1) Performance

**Fig 5:**
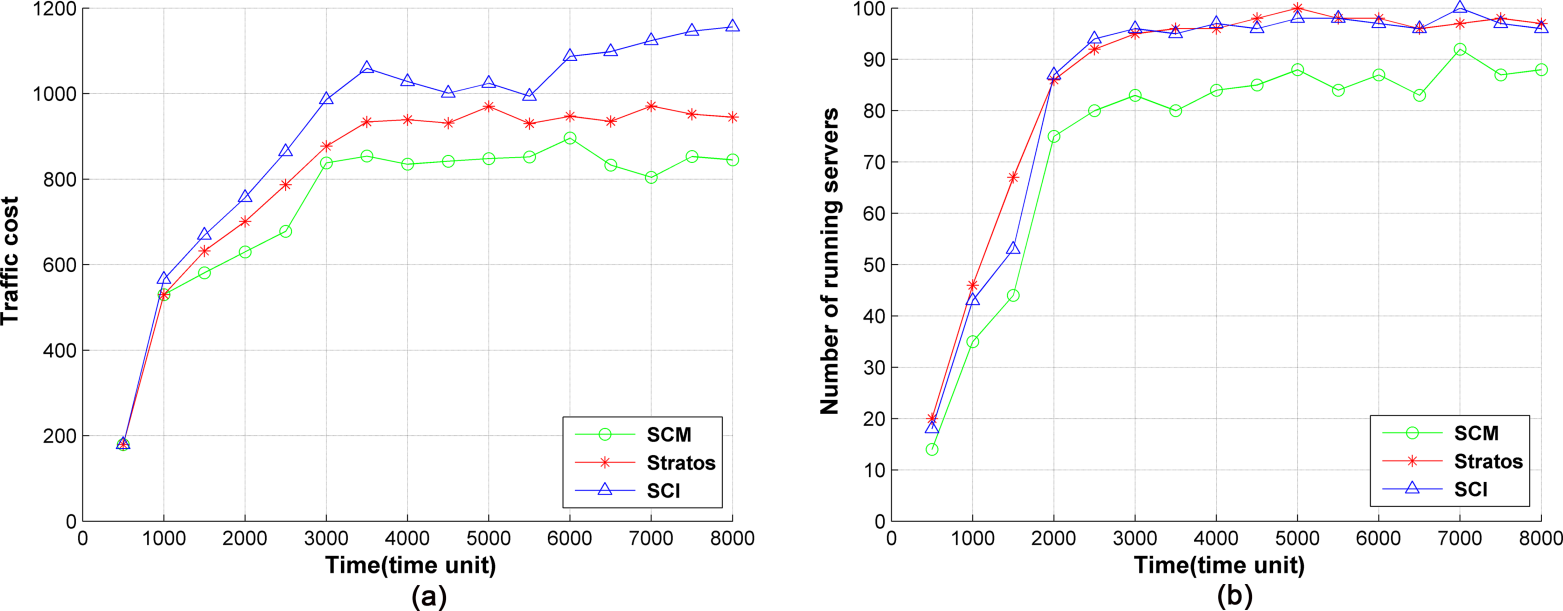
Comparison of traffic cost and number of running servers among the three methods.

In this section, service chain deployment methods, SCI [38] and Stratos [9], are compared with SCM in terms of traffic cost and number of running servers. In the experiments, the migration period is set to 500 time units, and the policy cost demand $d_{p}^{c}$ is defined as the maximum path cost in the network. We set *α*_1_ = 0.5, *α*_1_ = 0.3, and *α*_1_ = 0.2. The comparison of the traffic cost of the three methods is shown in Fig 5(A). In all three cases, the traffic cost of network increases in the beginning, which can be explained by the increasing number of the policies deployed in the network. We can see that the traffic cost of SCI is the highest among the three methods, since the traffic cost is not considered in SCI. By contrast, Stratos minimizes the traffic cost when server chains are deployed and the traffic cost is decreased effectively. However, in Stratos, the server chains cannot be moved once they are deployed. The layout of server chains will degenerate with the evolution of the network. Yet, SCM moves the server chains along with the evolution of the network and keeps low traffic cost. Therefore, the traffic cost of SCM is the lowest, about 20% lower than SCI and 10% lower than Stratos on average. The number of running servers with time for the three methods are shown in Fig 5(B). SCM has the lowest number of running servers. This is because SCM moves the server chains to reduce the number of running servers for energy saving. As can be seen, almost all the servers are running after 3000 time units when SCI or Stratos is used. The number of running servers is decreased by about 15% in SCM.

#### 2) Optimization of traffic cost

**Fig 6:**
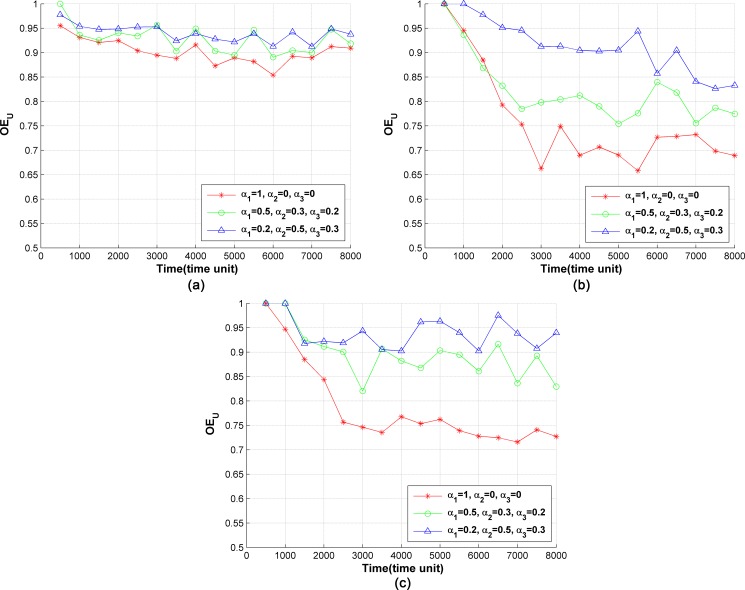
Optimization of network traffic cost by SCM. (a) *w*_*s*_ = 5, (b) *w*_*s*_ = 10, (c) *w*_*s*_ = 15.

The service chains are periodically migrated to optimize the layout of service instances. The migration period is 500 time units. The policy cost demand $d_{p}^{c}$ is defined as the maximum path cost in the network. Fig 6 shows the curves of *OE*_*U*_ with respect to time and different server capacities. With the network evolution, the number of policies deployed in the network rises at first, and then keeps stable. At the initial period of the network (Fig 6(A)), the ability of the traffic cost optimizaton is limited, and *OE*_*U*_ is close to 1 due to the small number of deployed policies. *OE*_*U*_ decreases with the increase of the number of policies in the network. This is because SCM migrates the service chains along with network evolution to decrease the traffic cost. The higher the weight of traffic cost *α*_1_ in the utility function (Eq (1)) is, the more significantly SCM optimizes the traffic cost. Therefore, *OE*_*U*_ is minimized when *α*_1_ = 1 and maximized when *α*_1_ = 0.2. As shown in Fig 6(A) and 6(B), when the weights are the same, *OE*_*U*_ at *w*_*s*_ = 10 is smaller than that when *w*_*s*_ = 5. The reason is that when the capacity of the server is enhanced, there are more resources in the network for server chain migration. Thus, better results can be achieved. When *w*_*s*_ = 15, there are a lot of idle server capacity resources. The layout of the service instances is already optimized when deploying service instances; therefore, *OE*_*U*_ is not significantly reduced compared with that when *w*_*s*_ = 10.

#### 3) Optimization of energy consumption

**Fig 7:**
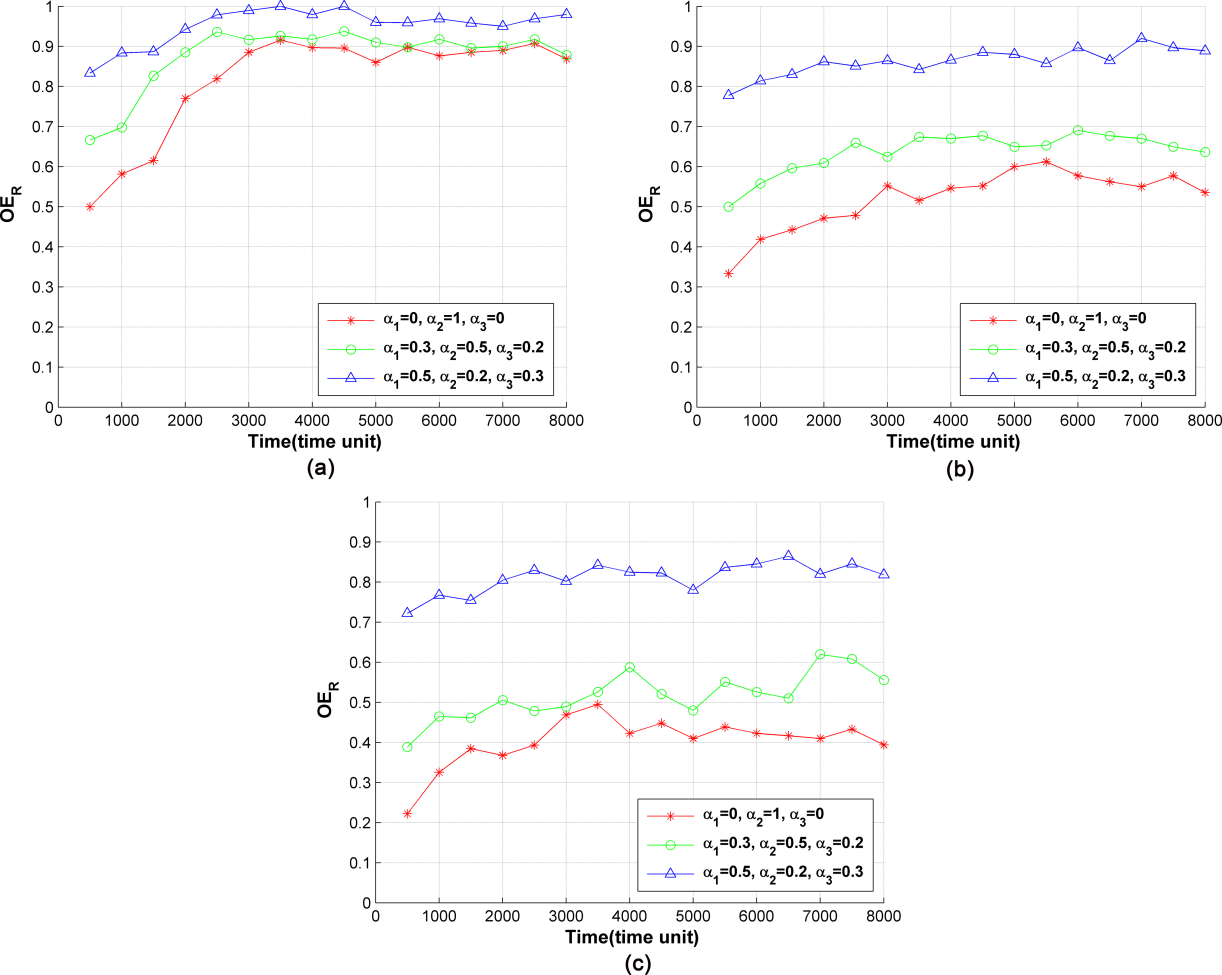
Optimization of the number of running servers by SCM. (a) *w*_*s*_ = 5, (b) *w*_*s*_ = 10, (c) *w*_*s*_ = 15.

The optimized rate of number of running servers *OE*_*R*_ is used to evaluate the optimization of energy consumption. When there is no service instance running on a server, this server will be turned off to save energy. The service chains are migrated with a period of 500 time units and the demand of cost $d_{p}^{c}$ is defined as the maximum path cost in the network. Fig 7 shows the curves of *OE*_*R*_ under different server loads. As shown in Fig 7(A), at the initial period, the number of deployed policies is small, and the server resources are sufficient. Therefore, the value of *OE*_*R*_ is low. With the increase of the number of policies, *OE*_*R*_ increases, as it becomes difficult to gather all the service instances in a small number of servers. The higher the weight *α*_2_ in the utility function is, the more significantly SCM optimizes the number of running servers. Therefore *OE*_*R*_ is minimized at *α*_2_ = 1 and maximized at *α*_2_ = 0.2. With the enhancement of server capability, more service instances can be held in each server, thus fewer servers can hold the same number of policies. Therefore, a larger *w*_*s*_ leads to a smaller *OE*_*R*_, as shown in Fig 7(A), 7(B) and 7(C).

#### 4) Optimization of service instance migration cost

**Fig 8:**
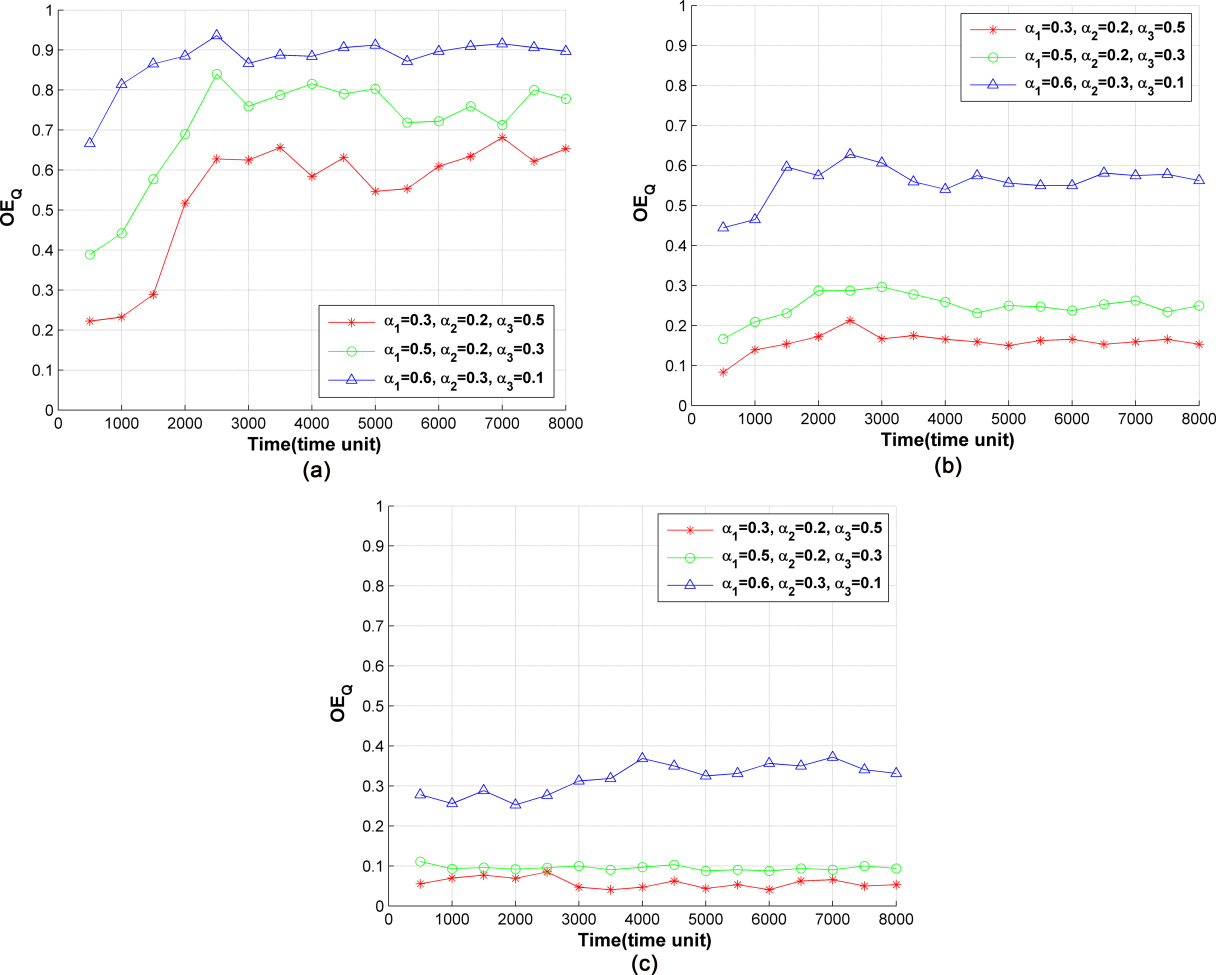
Proportion of migrated service instances in SCM. (a) *w*_*s*_ = 5, (b) *w*_*s*_ = 10, (c) *w*_*s*_ = 15.

The migration rate of service instance *OE*_*Q*_ is used to evaluate the cost of migration in SCM. The curves of *OE*_*Q*_ are shown in Fig 8 under different values of *w*_*s*_ and weight parameters. As shown in Fig 8(A), within 0–3000 time units, the value of *OE*_*Q*_ increases gradually with the increase of the number of deployed policies. After 3000 time units, when the number of policies stabilizes, *OE*_*Q*_ does as well. With the increase of the number of deployed policies, the amount of idle resources in the server declines. Because of the resource competition, the migration of a single service instance may lead to the re-layout of multiple service instances, leading to the increase of *OE*_*Q*_. When *α*_3_ in Eq (1) increases, the effect of *Q* on *Utility* is enhanced. Therefore, *OE*_*Q*_ tends to decrease with the increase of *α*_3_. Thus, *OE*_*Q*_ is maximized at *α*_3_ = 0.1 and minimized at *α*_3_ = 0.5 as shown in Fig 8(A), 8(B) and 8(C). When the capabilities of the servers are enhanced, the amount of idle resources of servers increases and the resource competition is less intense, resulting in a decrease in the value of *OE*_*Q*_. Thus, when the weights are equal, *OE*_*Q*_ decreases with an increase in the value of *w*_*s*_, as shown in Fig 8.

#### 5) Efficiency of PSO

In PSO, solutions may exist which do not satisfy the constraints. Eq (26) is used in SCM to update the particles’ positions to decrease the number of infeasible solutions. Let *N*_*I*_ be the number of infeasible solutions generated in the optimization. The occurrence probability of infeasible solutions can be calculated by Eq (32).

$$P\left(N_{I}\right)=\frac{N_{I}}{N_{P}\times MI}$$ (32)

**Fig 9:**
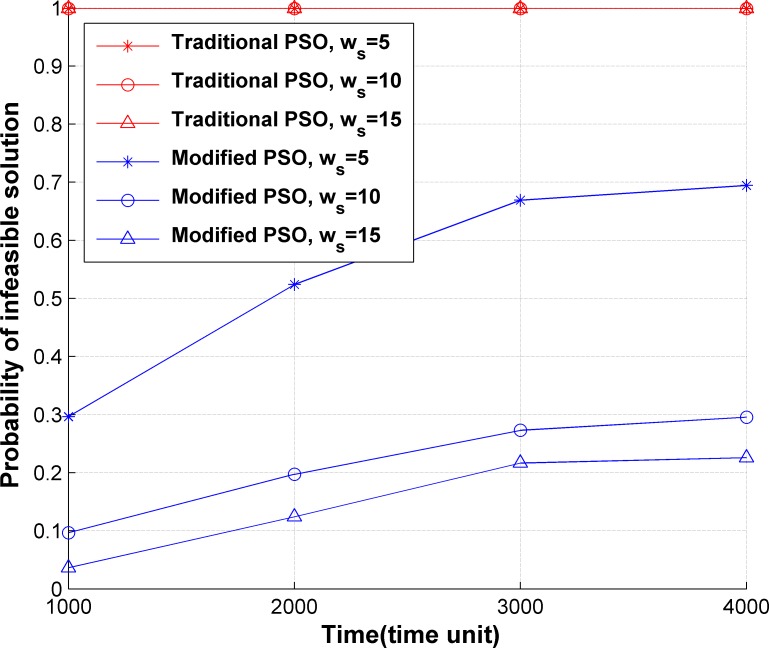
Comparison of occurrence probability of infeasible solutions between traditional PSO and modified PSO.

The comparison of *P*(*N*_*I*_) between the traditional PSO and our modified PSO is shown in Fig 9, where the x-axis represents the network operation time, and y-axis represents *P*(*N*_*I*_). The weights in the optimization target are *α*_1_ = 0.5, *α*_2_ = 0.3, *α*_3_ = 0.2. As shown in Fig 9, it is difficult for traditional PSO to find feasible solutions, because it is difficult to obtain solutions meeting the constraint in Eq (13) in traditional PSO due to the randomness in updating particle locations. SCM avoids the occurrence of numerous infeasible solutions using a modified PSO, which improves the optimization efficiency. At the early stage, a small number of policies are deployed in the network, so that there are enough server resources for deploying server chains. Hence, the constraints can be easily satisfied, and the occurrence probability of infeasible solutions is low. With an increasing number of policies in the network, server resources become tight and the occurrence probability of infeasible solutions increases. The larger *w*_*s*_ is more likely to meet the server capacity constraint. Therefore, the occurrence probability of infeasible solutions decreases when *w*_*s*_ increases.

#### 6) Efficiency of encouragement function

The encouragement function tends to migrate the instances in low-load servers to reduce the number of running servers. Let *R*_*m*_ and *R*_*m*_′ be the number of running servers after migration with and without the encouragement function, respectively. The reduction rate of number of running servers *rRate* is proposed, which can be calculated by Eq (33).

$$rRate=1-\frac{R_{m}}{R_{m}{}^{\prime }}$$ (33)

**Fig 10:**
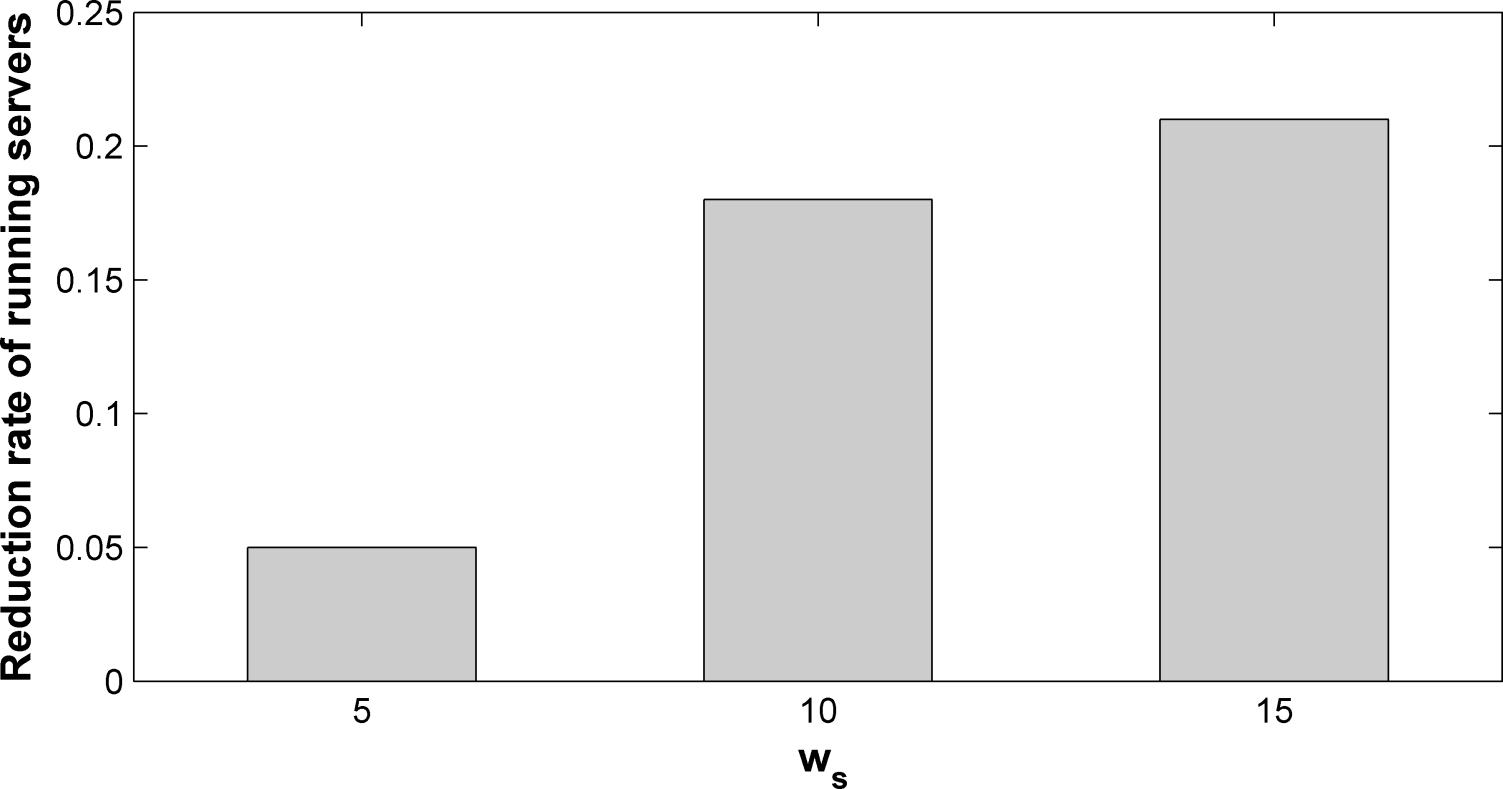
Reduction rate of number of running servers when encouragement function is used with different *w*_*s*_.

In this experiment, we deploy 40 policies in the network. The weights in the utility function are *α*_1_ = 0.5, *α*_2_ = 0.3, *α*_3_ = 0.2. Fig 10 shows the influence of the encouragement function to the number of running servers, where the x-axis represents server capacity, and y-axis represents *rRate*. Clearly, the number of running servers decreases when the encouragement function is used (*rRate* > 0). The encouragement function encourages service instances to migrate out of small-load servers. Thus, these service instances tend to be gathered to less servers, and the number of running servers decreases. The larger *w*_*s*_ is, the number of running servers will be reduced by a larger amount. This can be explained by the fact that the server capacity is the bottleneck against reducing the number of running servers, when *w*_*s*_ is small. With the increase of *w*_*s*_, the optimization of the encouragement function is more significant. When *w*_*s*_ ≥ 10, the encouragement function brings about a 20% reduction of the number of running servers.

## Conclusions and prospects

In the network deployed with services, the service layout may become inefficient with network evolution, resulting in a waste of network resources and energy. In this paper, we show two typical scenarios to illustrate the inefficiency of network resource deployment with network evolution. Regarding this problem, the SCM method is proposed to optimize the deployment of network resources. SCM migrates service chains and adjusts network resource allocation with the consideration of network performance, resource constraints, and instance migration cost. We model SCM as an ILP problem and solve it via modified PSO. An SCM prototype system, FlowMover, is designed based on the SDN controller. Our experiments show that SCM can reduce the traffic cost and energy consumption effectively. SCM is designed to increase the efficiency of the service instance layout, but the service type is ignored. However, different types of service have different requirements to networks. For example, IPTV services are sensitive to the network delay while file transmission services are sensitive to the bit error rate. In future work, service type will be considered in service chain migration for a better service quality.
